# Exploring the Role of NCX1 and NCX3 in an In Vitro Model of Metabolism Impairment: Potential Neuroprotective Targets for Alzheimer’s Disease

**DOI:** 10.3390/biology12071005

**Published:** 2023-07-14

**Authors:** Alessandra Preziuso, Silvia Piccirillo, Giorgia Cerqueni, Tiziano Serfilippi, Valentina Terenzi, Antonio Vinciguerra, Monia Orciani, Salvatore Amoroso, Simona Magi, Vincenzo Lariccia

**Affiliations:** 1 Department of Biomedical Sciences and Public Health-Pharmacology, School of Medicine, University “Politecnica delle Marche”, Via Tronto 10/A, 60126 Ancona, Italy; a.preziuso@pm.univpm.it (A.P.); s.piccirillo@staff.univpm.it (S.P.); g.cerqueni@staff.univpm.it (G.C.); t.serfilippi@pm.univpm.it (T.S.); s1095027@studenti.univpm.it (V.T.); a.vinciguerra@staff.univpm.it (A.V.); s.amoroso@staff.univpm.it (S.A.); v.lariccia@staff.univpm.it (V.L.); 2 Department of Clinical and Molecular Sciences-Histology, School of Medicine, University “Politecnica delle Marche”, Via Tronto 10/A, 60126 Ancona, Italy; m.orciani@staff.univpm.it

**Keywords:** NCX, energy metabolism impairment, Alzheimer’s disease, oxidative stress, calcium homeostasis

## Abstract

**Simple Summary:**

Alzheimer’s disease (AD) is a progressive neurodegenerative disorder and represents the most common cause of dementia among elderly people. It is characterized by the deterioration of brain cells and is linked to problems with energy production, cell metabolism, and harmful oxidative stress. Our study focused on two proteins called NCX1 and NCX3, which play a role in controlling calcium and sodium levels in cells. We wanted to understand whether these proteins may be involved in AD development when brain cells are exposed to metabolic impairment. To investigate this, we used a laboratory cell model and treated the cells with a substance called glyceraldehyde (GA) to mimic the metabolic dysfunction seen in AD. We used a technique called RNA interference to silence the expression of either NCX1 or NCX3 in the cells. We found that when NCX3 was silenced, the cells showed improved viability, increased energy production, and reduced damage from oxidative stress. Additionally, the levels of abnormal proteins associated with AD, such as Aβ and pTau, were decreased. However, silencing NCX1 did not have the same positive effects, except for increased energy production. These findings suggest that targeting NCX3 may be a potential strategy to prevent the development of AD associated with metabolic dysfunction. Considering the paucity of pharmacological therapies, this knowledge could be valuable for identifying new potential treatments for AD.

**Abstract:**

Alzheimer’s disease (AD) is a widespread neurodegenerative disorder, affecting a large number of elderly individuals worldwide. Mitochondrial dysfunction, metabolic alterations, and oxidative stress are regarded as cooperating drivers of the progression of AD. In particular, metabolic impairment amplifies the production of reactive oxygen species (ROS), resulting in detrimental alterations to intracellular Ca^2+^ regulatory processes. The Na^+^/Ca^2+^ exchanger (NCX) proteins are key pathophysiological determinants of Ca^2+^ and Na^+^ homeostasis, operating at both the plasma membrane and mitochondria levels. Our study aimed to explore the role of NCX1 and NCX3 in retinoic acid (RA) differentiated SH-SY5Y cells treated with glyceraldehyde (GA), to induce impairment of the default glucose metabolism that typically precedes Aβ deposition or Tau protein phosphorylation in AD. By using an RNA interference-mediated approach to silence either NCX1 or NCX3 expression, we found that, in GA-treated cells, the knocking-down of NCX3 ameliorated cell viability, increased the intracellular ATP production, and reduced the oxidative damage. Remarkably, NCX3 silencing also prevented the enhancement of Aβ and pTau levels and normalized the GA-induced decrease in NCX reverse-mode activity. By contrast, the knocking-down of NCX1 was totally ineffective in preventing GA-induced cytotoxicity except for the increase in ATP synthesis. These findings indicate that NCX3 and NCX1 may differently influence the evolution of AD pathology fostered by glucose metabolic dysfunction, thus providing a potential target for preventing AD.

## 1. Introduction

Alzheimer’s disease (AD) is a progressive neurological disorder, and by far is considered the most common form of dementia in the elderly [1]. Despite the impressive effort in deciphering cause-and-effect trajectories at work, AD pathogenesis is still ill-defined and, thereby, its treatment remains a major healthcare challenge [2]. AD is characterized by irreversible memory loss and cognitive impairment along with widespread neuronal degeneration. The pathognomonic hallmarks of AD are senile plaques, primarily composed by insoluble aggregates of amyloid β (Aβ) peptides, and neurofibrillary tangles consisting of the hyperphosphorylated form of Tau protein (pTau). AD has a complex and multifactorial nature [3], in which various molecular and cellular pathways cooperate to shape its course [4,5]. Bioenergetic dysfunction can be considered as the common underlying thread that links the many dimensions of AD pathology. An adequate glucose metabolism is important to support neuronal activity and guarantee cognitive resilience of healthy aging [6], whereas decreases in brain glucose uptake take place during the preclinical stage of AD and contribute to the onset and progression of the disease [7,8,9,10,11]. Interestingly, dysregulations of the glycolytic flux are typically observed in AD patients [12], being associated with the increase in the deposition of senile plaques and neurofibrillary tangles and cognitive decline [13]. In this framework, metabolic impairment will feed the generation of reactive oxygen species (ROS) and disrupt cellular Ca^2+^ homeostasis, thus creating a self-reinforcing loop of neurodegeneration. In this line, it has been recently demonstrated that, in rat cortical neurons spurred with glyceraldehyde (GA) to alter glucose metabolism [14], an antioxidant treatment improves cell viability by: (i) reducing the increase in Aβ and pTau levels, (ii) recovering the reduction of ATP content, and (iii) counteracting the perturbation of cellular Ca^2+^ homeostasis within both cytoplasmic and mitochondrial compartments [14]. Given the crucial role of Ca^2+^ in the regulation of neuronal physiology at multiple levels, it is not surprising that even subtle perturbation of Ca^2+^ homeostasis may dramatically challenge neuronal functions and survival [15,16,17]. On the one hand, dysregulation in Ca^2+^ signaling influences Aβ aggregation and deposition and Tau phosphorylation [18], and in more general terms, incites the longitudinal progression of AD pathology [19]. On the other hand, activation of the amyloidogenic pathway has the potential to remodel neuronal Ca^2+^ signaling and alter Ca^2+^ homeostasis, contributing to learning and memory impairment [20,21,22,23,24]. Therefore, restoring a balanced control over Ca^2+^ homeostasis may promote survival of neurons that would otherwise succumb to AD injury.

Among the ionic transporters that provide significant contributions to the Ca^2+^ homeostasis of brain cells there is the Na^+^/Ca^2+^ exchanger (NCX). In particular, the NCX plays a critical role in regulating Ca^2+^ and Na^+^ homeostasis at both plasma membrane and mitochondrial levels [25]. As a secondary active transport, the NCX can operate either in the forward mode (Na^+^ influx/Ca^2+^ efflux) or in the reverse mode (Na^+^ efflux/Ca^2+^ influx) depending on the electrochemical ion gradients [26]; although, exchange activity proceeds under constant control of redundant regulatory inputs [27]. Three isoforms have been described, NCX1, NCX2, and NCX3, which are differentially expressed in a tissue-specific manner [28]. The ionic and electrical outputs generated by NCX proteins are modified by and can influence the evolution of pathological paradigms in many cell types [29]. In particular, interest has been shown in exploring their functions in AD pathology [30], but the specific role(s) played by NCXs in the metabolic derangements that incite AD evolution remain(s) poorly defined.

As far as NCX1 and NCX3 are concerned, these two exchangers are key determinants of intracellular Ca^2+^ homeostasis in excitable cells, like neurons and cardiomyocytes, especially in pathological settings where energy balance is significantly compromised [26]. In the past few years, significant attention has been focused on NCX3, since reductions of its activity or expression have been associated with poor cell survival in different models of excitotoxicity-induced cell damage and cerebral ischemia [30,31,32,33,34,35]. Interestingly, our previous studies revealed that, in energy metabolism-compromised states like cardiac and neuronal hypoxia/reoxygenation injury and neurodegenerative models such as AD and Parkinson’s disease (PD), NCX1 plays an essential role for the metabolic use of glutamate to sustain mitochondrial ATP production and guarantee improved survival [36,37,38]. However, it has been also observed that in a setting of cardiac ischemia/reperfusion (I/R) injury, NCX1 can play a dual role since its inhibition during I/R is crucial for cell survival, while its activity is strongly involved in providing protection during ischemic preconditioning [39]. Therefore, NCX isoforms may exert diverse roles that strictly depend on the specific pathologic conditions under which the exchanger works [29,32,40,41].

In light of this evidence and the fact that alteration of Ca^2+^ handling is a central driver of AD progression, in the present study, we aimed to elucidate the role of NCX1 and NCX3 in an in vitro model of retinoic-acid (RA) differentiated SH-SY5Y cells, where the exposure to GA induces a glucose metabolism impairment [14,36]. The hypometabolism condition that this model simulates is associated with mitochondrial dysfunction and redox imbalance that are typical early alterations of AD.

## 2. Materials and Methods

### 2.1. Cell Culture and Treatments

The SH-SY5Y cell line, derived from human neuroblastoma, was purchased from the American Type Culture Collection (CRL-2266). The cells were cultivated in Dulbecco’s Modified Eagle Medium (DMEM; Corning, New York, NY, USA) supplemented with 10% fetal bovine serum (FBS, Corning), 100 U/mL penicillin, and 100 µg/mL streptomycin (Corning). They were maintained in an incubator at 37 °C with a 5% CO_2_ atmosphere. In order to induce their differentiation into neuron-like cells, they were exposed to 10 µM all-trans retinoic acid (RA) for 6 days. Prior to treatment with GA for 24 h, the cells were initially subjected to siRNA-mediated silencing (as described in the subsequent paragraph) [14]. Furthermore, alongside GA, the cells were treated with SN6, a compound known as 2-[[4-[(4Nitrophenyl) methoxy] phenyl] methyl]-4-thiazolidinecarboxylic acid ethyl ester (3 µM) for 24 h. At the end of the experimental protocol, the cells were collected for subsequent analysis.

### 2.2. Silencing of NCX1 and NCX3

To knock-down the expression of NCX1 and NCX3 in SH-SY5Y cells, RNA interference (RNAi) technique was performed. The HiPerfect Transfections Kit from Qiagen (Hilden, Germany) was used along with FlexiTube siRNA designed for NCX1 (Qiagen, Hs_SLC8A1_9, 5′-CAGGCCATCTTCTAAGACTGA-3′) and FlexiTube siRNA for NCX3 (Qiagen, Hs_SLC8A3_7, 5′- ACCATTGGTCTCAAAGATTCA-3′) [40]. It should be noted that NCX2 is not expressed in either the undifferentiated or RA-differentiated states of SH-SY5Y cells [42], making them a valuable tool to focus on NCX1 and NCX3. In brief, following RA differentiation, SH-SY5Y cells were incubated with siRNA oligonucleotides and transfection reagents for a period of 48 h. Once the silencing process was complete, the cells were exposed to specific treatments, and cell viability, ATP content, mitochondrial ROS production, AD biomarkers, and NCX activity were evaluated.

### 2.3. Viability Assay

Cell viability was assessed by the MTT assay and by determining the amount of lactate dehydrogenase (LDH) released from damaged cells into the culture medium. The MTT assay evaluates the capacity of mitochondria to metabolize the yellow tetrazolium salt 3-(3,4-dimethylthiazol-2-yl)-2,5-diphenyltetrazolium bromide (MTT) resulting in the formation of insoluble purple formazan crystals. Following incubation with MTT solution (0.5 mg/mL in PBS) for 1 h in a dark environment at 37 °C and 5% CO_2_, the formazan crystals were dissolved in dimethyl sulfoxide (DMSO). The quantity of formazan generated was directly proportional to the mitochondrial activity of the cells, and the absorbance value was measured at a wavelength of 540 nm employing a Victor Multilabel Counter plate reader from Perkin Elmer (Waltham, MA, USA). A decline in mitochondrial activity led to a decrease in the absorbance value observed. As for LDH activity, following the experimental procedure, 50 µL of the culture medium were collected and transferred to a 96-well plate. Then, the medium was incubated with the Cytotoxicity Detection Kit reaction mixture from Roche (Basilea, Switzerland) for a duration of 30 min. Subsequently, the absorbance was read at 490 nm using a Victor Multilabel Counter plate reader. The obtained results were expressed as percentages in relation to the control value.

### 2.4. ATP Assay

Intracellular ATP levels were measured using a luciferase-luciferin system (ATPlite, Perkin Elmer, Waltham, MA, USA) [36]. SH-SY5Y were plated and differentiated onto a 96-well View Plate from Perkin Elmer. At the end of indicated treatments, the cells were lysed, and the ATP assay was carried out as per manufacturer’s instructions. The levels of ATP were quantified utilizing a luminescence-based Victor Multilabel Counter plate reader from Perkin Elmer. These values were then normalized to the corresponding protein content. The results were expressed as a percentage relative to the control value.

### 2.5. Evaluation of Mitochondrial ROS Production

Mitochondrial ROS levels were determined using MitoTracker CM-H2XRos (Invitrogen Life Technologies, Carlsbad, CA, USA), as previously described [36]. RA-differentiated SH-SY5Y cells were seeded onto coverslips and subjected to the experimental protocol. Subsequently, the cells were loaded with a dye concentration of 300 nM for a duration of 30 min at 37 °C. Confocal images were obtained using a 510 LSM microscope (Carl Zeiss, Milan, Italy) equipped with a META detection system. For visualization of MitoTracker CM-H2XRos, the dye was excited at 560 ± 10 nm, and its emission was measured at 620 ± 20 nm. Images were captured at 5 s intervals, allowing for the monitoring of basal ROS levels over approximately 200 s. Following image acquisition, the fluorescence intensity was analyzed offline. The fluorescence values were expressed as a percentage relative to the control value.

### 2.6. Immunofluorescence Experiments

#### 2.6.1. Primary Antibodies

Aβ_1–42_ peptides were detected using a mouse monoclonal IgG1 antibody (clone 12F4, Cat. 805501, Biolegend, San Diego, CA, USA, dilution 1:100 in PBS with 1% BSA). pTau protein was evaluated by using a human PHF-Tau monoclonal IgG antibody (clone AT100, Cat. MN1060, recognizing Thr212 and Ser214, Thermo Scientific, Milano, Italy, dilution 1:1000 in PBS with 1% BSA). NCX1 protein was detected by using a rabbit polyclonal antibody (π 11-13, Swant, Switzerland, dilution 1:100 in PBS with 1% BSA). The expression of NCX3 protein was evaluated by using a rabbit polyclonal antibody (95209, Swant, Switzerland, dilution 1:100 in PBS with 1% BSA).

#### 2.6.2. Immunofluorescence Staining

Following the experimental procedures, cells were exposed to 300 nM MitoTracker Red CMXRos (excitation/emission wavelengths: 579/599 nm) (Invitrogen Life Technologies) for 30 min, at 37 °C. Subsequently, the cells were fixed using 3.7% formaldehyde in phosphate-buffered saline at room temperature (RT) for 30 min, followed by permeabilization with 0.1% Triton X-100 at RT for 5 min [36]. Afterwards, the cells were incubated with primary antibodies (Aβ_1–42_, pTau AT100, and NCX1-3) for 1 h at RT, and then treated with Alexa anti-mouse 488 conjugated secondary antibody, (excitation/emission wavelengths: 499/520 nm) (Cat. A11059 Thermo Scientific, diluted 1:200), to visualize immunoreactivity. Protein fluorescence images were acquired using the LSM 510 confocal system from Carl Zeiss at 5 s intervals, the fluorescence values were monitored for approximately 20 s. Following image acquisition, the fluorescence intensity was analyzed offline, and the resulting values were expressed as percentages relative to the control value.

### 2.7. NCX Activity

#### 2.7.1. Analysis of NCX Activity

Solutions: Ca-PSS was composed of (in mM): 140 NaCl; 5 KCl; 1 MgCl_2_; 10 glucose; 2 CaCl_2_; and 20 HEPES, buffered to pH 7.4 with Tris. Na-PSS (Ca^2+^-free solution) was composed of (in mM): 140 NaCl; 5 KCl; 1 MgCl_2_; 10 glucose; 0.1 EGTA; and 20 HEPES, buffered to pH 7.4 with Tris. K-PSS was composed of (in mM): 140 KCl; 1 MgCl_2_; 10 glucose; 0.1 CaCl_2_; and 20 HEPES, buffered to pH 7.4 with Tris.

#### 2.7.2. Experimental Protocol

The levels of intracellular Ca^2+^ were determined using an LSM 510 confocal system from Carl Zeiss through computer-assisted video imaging on a single-cell basis. SH-SY5Y cells were treated onto 25 mm coverslips according to the experimental protocol. Following that, the cells were loaded with 4 µM Fluo-4/AM (Molecular Probe, Eugene, OR, USA) dissolved in Ca-PSS supplemented with 0.08% pluronic acid (Molecular Probe) for 40 min in darkness at RT. Cells were rinsed once with Na-PSS and subsequently treated with 1 μM thapsigargin in Na-PSS for 10 min [40]. By switching Na-PSS to K-PSS, we assessed the uptake of Ca^2+^ via the reverse mode. A peristaltic pump was used to change bath solutions, and images were captured every 5 s. A heated microscope stage and climate box from PeCon GmbH were employed to maintain the cells and perfusion solutions at 37 °C. An argon laser at 488 nm provided the excitation light, and the emission in the range of 505–530 nm was recorded in a time-lapse manner. Following image acquisition, we conducted the fluorescence intensity analysis offline, following the previously described methodology [40].

### 2.8. Drugs and Chemicals

SN6 was obtained from Tocris (Bristol, UK). All other chemicals were of analytical grade and were purchased from Sigma (Milan, Italy).

### 2.9. Data Analysis

Data were reported as the mean ± standard error of the mean (S.E.M.). GraphPad Prism^®^ 5 software (San Diego, CA, USA) was used for the statistical analyses of the results. One-way ANOVA analysis followed by Dunnett’s post hoc test were applied to determine the differences between the experimental groups. Differences were considered statistically significant when *p* < 0.05.

## 3. Results

### 3.1. Role of NCX1 and NCX3 in the Perturbation of Cell Viability and ATP Production in GA-Challenged Cells

**Figure 1:**
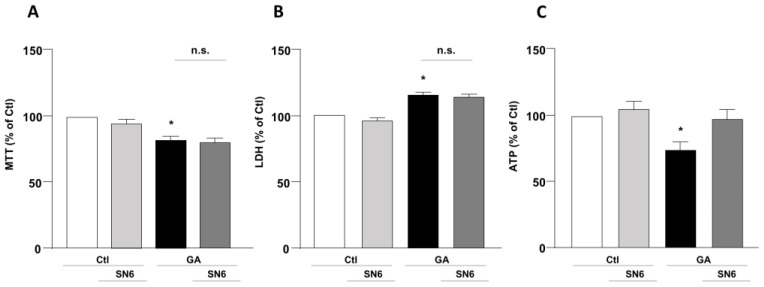
Effect of SN6 on cell viability and intracellular ATP levels in RA-differentiated SH-SY5Y cells challenged with GA. (**A**) Effect of SN6 (3 µM) on cell viability assessed by MTT assay; (**B**) extracellular LDH release; and (**C**) ATP production. The cells were exposed to a combination of SN6 (3 µM) and GA (1 mM) for a duration of 24 h. In each experiment, MTT reduction, extracellular LDH release, and ATP levels were expressed as percentage of the control. Each column represents the mean ± S.E.M. of at least 3 experiments performed in triplicate. Significant differences were evaluated by one-way ANOVA followed by Dunnett’s post hoc test. A F (3, 16) = 9.330. * Significant vs. control groups (*p* < 0.01 vs. Ctl, *p* < 0.05 vs. SN6). B F (3, 14) = 20.44. * Significant vs. control groups (*p* < 0.001 vs. Ctl, *p* < 0.0001 vs. SN6). C F (3, 20) = 5.318. * Significant vs. all groups (*p* < 0.05 vs. Ctl and GA + SN6, *p* < 0.01 vs. SN6). Ctl control, GA glyceraldehyde, n.s. not significant.

**Figure 2:**
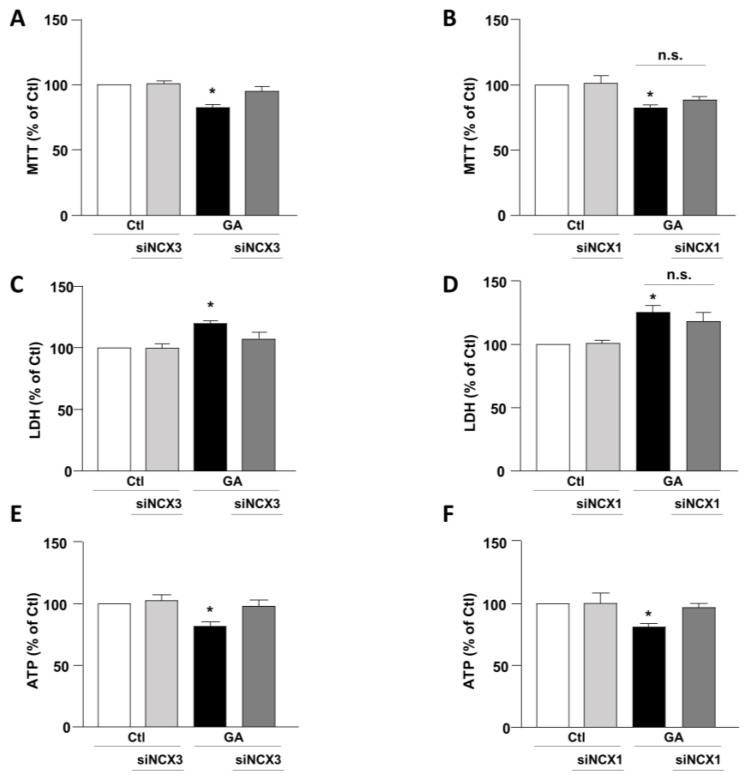
Effect of NCX1 and NCX3 silencing on cell survival and intracellular ATP levels in RA-differentiated SH-SY5Y cells exposed to GA. Following the silencing of either NCX3 or NCX1, the cells were subjected to GA treatment (1 mM) for 24 h. Cell viability results, as determined by MTT assay (**A**,**B**) or LDH assay (**C**,**D**), and measurements of intracellular ATP levels (**E**,**F**) are graphed in the column charts and reported as percentages relative to the respective control group. Each column represents the mean ± S.E.M. of at least 4 experiments performed in triplicate. Significant differences were evaluated by one-way ANOVA followed by Dunnett’s post hoc test. A F (3, 18) = 11.72. * Significant vs. all groups (*p* < 0.001 vs. Ctl and siNCX3, *p* < 0.01 vs. and GA + siNCX3); B F (3, 22) = 6.147. * Significant vs. control groups (*p* < 0.01 vs. Ctl, *p* < 0.05 vs. siNCX3); C F (3, 16) = 8.034. * Significant vs. all groups (*p* < 0.01 vs. Ctl and siNCX3, *p* < 0.05 vs. GA + siNCX3); D F (3, 24) = 8.046. * Significant vs. control groups (*p* < 0.01 vs. Ctl and siNCX1). E F (3, 16) = 5.532. * Significant vs. all groups (*p* < 0.05 vs. Ctl and GA + siNCX3, *p* < 0.01 vs. siNCX3); F F (3, 18) = 6.503. * Significant vs. all groups (*p* < 0.01 vs. Ctl and siNCX1, *p* < 0.05 vs. GA + siNCX1). siNCX1 siRNA for NCX1, siNCX3 siRNA for NCX3.

To mimic hypometabolism associated with AD, RA-differentiated SH-SY5Y cells were exposed for 24 h to GA (1 mM), as previously described [14,36]. To obtain further insights on the involvement of the exchangers in this experimental model, we initially used a pharmacological approach based on the NCX inhibitor 2-[[4-[(4Nitrophenyl) methoxy] phenyl] methyl]-4-thiazolidinecarboxylic acid ethyl ester (SN6, 3 µM), which was added together with GA to the cells. At this concentration, SN6 is expected to inhibit NCX1, with a weak blocking effect on NCX3 [43]. Strikingly, we observed that GA-exposed cells to SN6 did not improve viability (Figure 1A,B), as assessed by both MTT and LDH assays (Figure 1A,B), while it significantly rescued the intracellular ATP levels to the control value (Figure 1C). To further dissect the specific role of NCX1 and NCX3, we then selectively silenced their expression by using an RNA interference (RNAi)-mediated approach [40]. Interestingly, while NCX1 silencing was not accompanied by any improvement in cell viability (Figure 2B,D), the knocking-down of NCX3 expression was able to fully counteract the negative effects of GA on cell viability (Figure 2A,C). Of note, the intracellular ATP levels were restored by both NCX1 and NCX3 silencing (Figure 2E,F).

### 3.2. NCX3 Silencing Reduced the Production of Mitochondrial ROS Induced by GA

**Figure 3:**
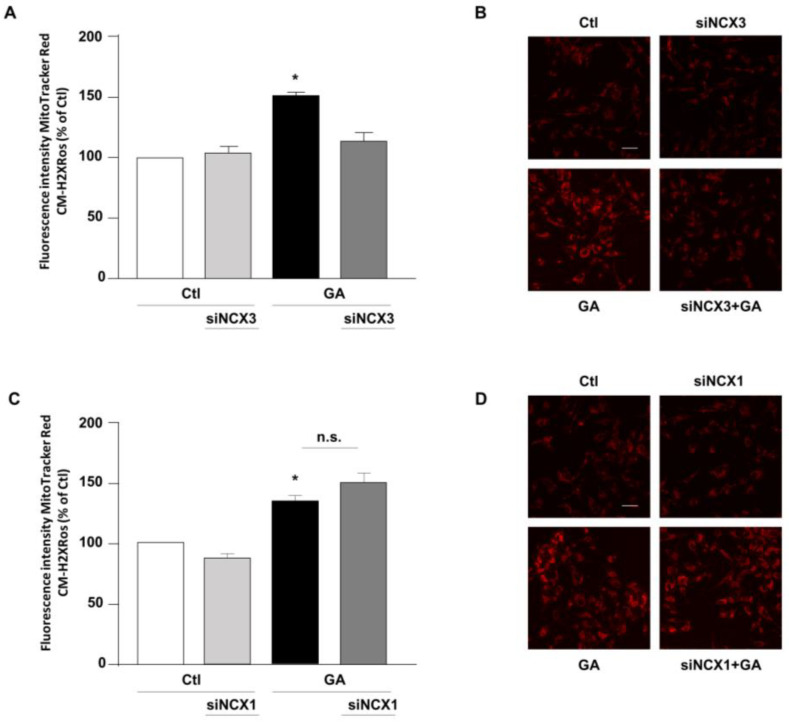
Effect of NCX3 and NCX1 silencing on the generation of ROS within the mitochondria of RA-differentiated SH-SY5Y cells challenged with GA. (**A**,**C**) MitoTracker Red CM-H2XRos fluorescence, indicative of mitochondrial ROS levels, was evaluated after silencing NCX3 (**A**) and NCX1 (**C**) in cells exposed to GA (1 mM) for 24 h and reported as percentages relative to the respective control group. Each column represents the mean ± S.E.M. of at least 3 experiments performed in triplicate. Images displaying mitochondrial ROS fluorescence levels are shown for NCX3 silencing (**B**) and NCX1 silencing (**D**) and are representative of at least 3 experiments (50–100 cells for each experimental group were analyzed). Scale bar 50 µM. Significant differences were evaluated by one-way ANOVA followed by Dunnett’s post hoc test. A F (3, 8) = 25.83. * Significant vs. all groups (*p* < 0.001 vs. Ctl and siNCX3, *p* < 0.01 vs. GA + siNCX3); C F (3, 11) = 14.28. * Significant vs. control groups (*p* < 0.05 vs. Ctl, *p* < 0.01 vs. siNCX1).

The above-mentioned findings suggested that contributions of NCXs to the GA-induced cellular alterations are isoform-specific, thus leading us to hypothesize that, even though both NCX1 and NCX3 are involved in the GA-induced decrease in the intracellular ATP content, their differential impacts on GA-induced cell injury rely on additional responses. Therefore, we sought to further explore the specific contribution of NCX1 and NCX3 to the various cellular alterations that underlie GA toxicity and that can predispose the cells to develop an AD-like phenotype [14]. Among those, we firstly considered the production of ROS. Our previous research demonstrates that GA increases the production of ROS at the mitochondrial level [14]. Therefore, we attempted to understand whether either NCX1 or NCX3 could have a role in this specific setting. As expected, GA increased mitochondrial ROS production (Figure 3). We found that by silencing NCX3, ROS levels were significantly reduced in cells challenged with GA (Figure 3A,B), while NCX1 silencing had no effect at all (Figure 3C,D). Under resting conditions, either NCX1 or NCX3 silencing did not produce any significant changes in the formation of ROS.

### 3.3. NCX3 Silencing Significantly Reduced Aβ and pTau Levels in RA-Differentiated SH-SY5Y Cells

**Figure 4:**
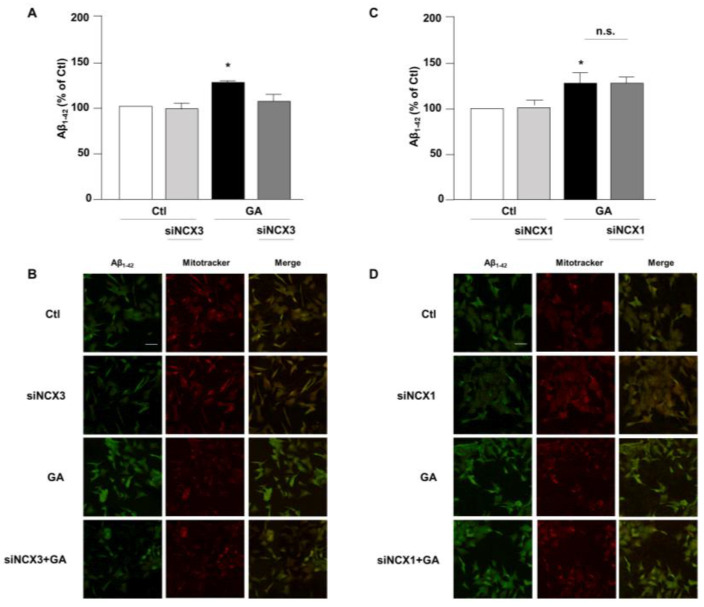
Evaluation of Aβ_1–42_ expression in RA-differentiated SH-SY5Y cells after knocking-down of NCX3 and NCX1 expression and GA treatment. (**A**,**C**) Quantitative analysis and (**B**,**D**) representative images depicting the expression of Aβ_1–42_. Scale bar 50 µM. Images were representative of at least 3 experiments (50–100 cells for each experimental group were analyzed). Differences among means were evaluated by one-way ANOVA followed by Dunnett’s post hoc test. A F (3, 11) = 21.36. (**A**,**C**) Each column represents the mean ± S.E.M. obtained from at least 4 experiments performed in triplicate. * Significant vs. all groups (*p* < 0.01 vs. Ctl and siNCX3, *p* < 0.05 vs. GA + siNCX3); C F (3, 11) = 8.238. * Significant vs. control groups (*p* < 0.01 vs. Ctl, *p* < 0.05 vs. siNCX1).

**Figure 5:**
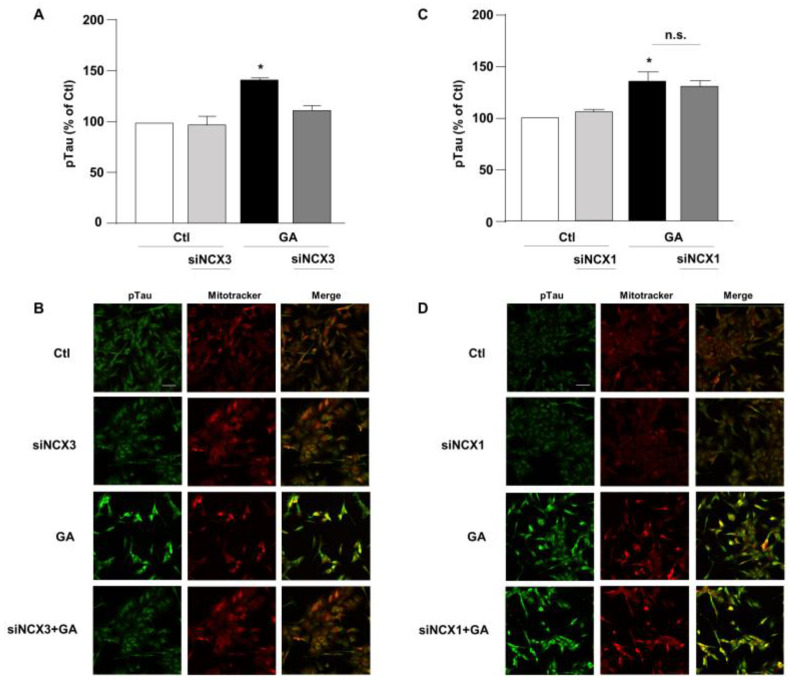
Evaluation of pTau expression in RA-differentiated SH-SY5Y cells after knocking-down of NCX3 and NCX1 expression and GA treatment. (**A**,**C**) Quantitative analysis and (**B**,**D**) representative images depicting the expression of pTau. Scale bar 50 µM. Each column represents the mean S.E.M. obtained from at least 3 experiments (50–100 cells for each experimental group were analyzed). Differences among means were evaluated by one-way ANOVA followed by Dunnett’s post hoc test. A F (3, 11) = 21.36. (**A**,**C**) Each column represents the mean ± S.E.M. of at least 3 experiments performed in triplicate. * Significant vs. all groups (*p* < 0.0001 vs. Ctl, *p* < 0.001 vs. siNCX3 and *p* < 0.05 vs. GA + siNCX3); C F (3, 11) = 8.238. * Significant vs. control groups (*p* < 0.01 vs. Ctl, *p* < 0.05 vs. siNCX1).

Accumulation of Aβ and hyperphosphorylated tau are characteristic lesions of AD brains. Findings from our previous studies show that when cells are treated with GA, the overall cellular injury is accompanied by sustained alterations of these two biomarkers [14,36]. Treatments that normalize both Aβ and pTau levels significantly improve cell viability [14], suggesting that such changes are negatively related to cell viability. Therefore, considering the different role exerted by NCX1 and NCX3 on the GA-induced cell damage, we sought to investigate whether this effect could be related to a different influence of NCX1 and NCX3 on the deposition of Aβ and/or on the phosphorylation of pTau. Interestingly, we observed that the knocking-down of NCX3 significantly reduced the accumulation of both Aβ (Figure 4A,B) and pTau, whose levels were significantly increased after GA challenge (Figure 5A,B). The silencing of NCX1 did not modify Aβ (Figure 4C,D) or pTau levels (Figure 5C,D), further adding ground to the distinct roles played by the two isoforms in a context of deranged metabolism.

### 3.4. NCX3 Silencing Normalizes NCX Activity after GA Challenge in RA-Differentiated SH-SY5Y Cells

**Figure 6:**
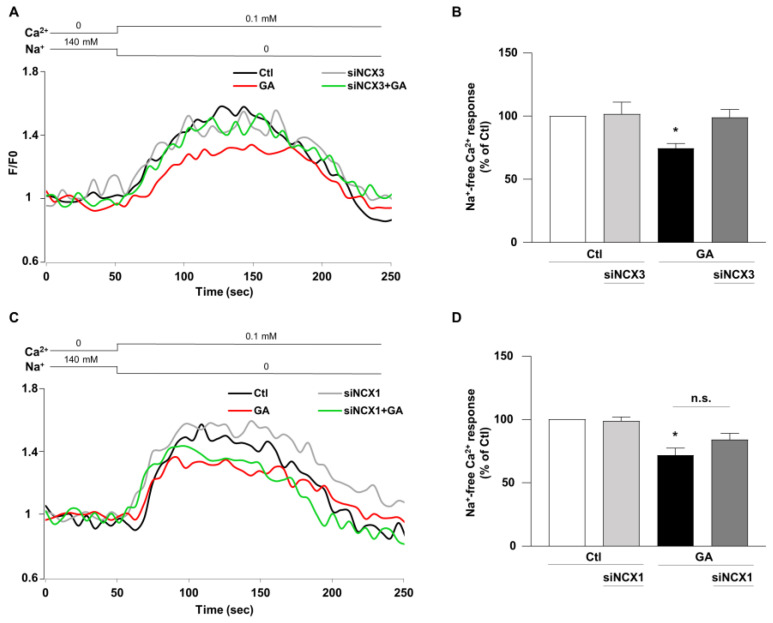
Effect of NCX3 and NCX1 knocking-down on NCX activity after GA challenge in RA-differentiated SH-SY5Y cells. (**A**,**C**) Representative records of Ca^2+^ response to Na^+^ free after incubation were obtained under different conditions: control (black line), siNCX1 and siNCX3 (grey line), GA treatment (red line) and GA treatment in combination with siRNA (siNCX3 and siNCX1, green line). (**B**,**D**) Quantitative analyses were performed at the end of the experimental protocol. The fluorescence intensity values were normalized to the resting fluorescence (F/F0), and the activity of NCX was expressed as the percentage increase in fluorescence (Δ% fluorescence increase). The bar plot represents the mean ± S.E.M. of the fluorescence increase induced by the Na^+^ free pulse. For each experimental group, Δ% values used for statistical analysis were derived from a minimum of 4 experiments performed in triplicate, with 100–150 cells recorded in each session. Each column represents the mean ± S.E.M. of at least 4 experiments performed in triplicate. Differences among means were evaluated by one-way ANOVA followed by Dunnett’s post hoc test. B F (3, 17) = 4.62. * Significant vs. all groups (*p* < 0.05 vs. Ctl, siNCX3 and GA + siNCX3). D F (3, 25) = 12.53. * Significant vs. control groups (*p* < 0.0001 vs. Ctl, *p* < 0.001 vs. siNCX1).

Once established that NCX1 and NCX3 might have a different role in controlling the overall cellular derangement induced by GA and considering that Ca^2+^ homeostasis can be significantly affected by the metabolic alterations induced in this specific experimental setting [14], we lastly examined the impact of NCX1 and NCX3 activities. The activity of the exchanger was assessed by measuring the uptake of Ca^2+^, which is dependent on the Na^+^ gradient in cells that were loaded with Fluo-4, using fluorescence signals as a monitoring tool [40]. During the analysis, we monitored the reverse-mode activity by gradually reducing the extracellular Na^+^ (replaced by K^+^) at the end of the experimental protocol. As summarized in Figure 6, when the NCX reverse mode was triggered, Ca^2+^ uptake was significantly lower (∼30%) in GA-treated cells rather than in controls, as revealed by the decrease in fluorescence signal. When NCX3 was silenced, NCX reverse-mode activity was recovered towards control values (Figure 6A,B), while the silencing of NCX1 was completely ineffective in restoring the exchanger activity (Figure 6C,D). Interestingly, neither NCX3 nor NCX1 silencing produced any significant change under control conditions. Furthermore, the observed changes in the NCXs activity were not accompanied by alterations in protein expression, in all the tested experimental conditions (Figure S1).

## 4. Discussion

Numerous evidence supports the multifactorial nature of AD. Over the years, it has been recognized that metabolic alterations are crucial for this neurodegenerative disorder. In this study we focused on the metabolic defects that may create an environment suitable for AD development by using an in vitro model mimicking the alteration of glucose metabolism in RA-differentiated SH-SY5Y cells [14,36,44]. To mimic bioenergetic alterations (i.e., hypometabolism, mitochondrial dysfunction and redox imbalance [14]), which are regarded as early pathological events of AD neurodegenerative spiral, we exposed cells to GA [14]. GA cytotoxicity is also related to the alteration of Ca^2+^ homeostasis [14,36], another element that significantly contributes in giving rise to a vicious cycle capable of increasing the levels of Aβ and pTau and of activating cell death pathways [24]. In line with this so called “Ca^2+^ hypothesis in AD”, we aimed to investigate the implication of NCX1 and NCX3 in a context of metabolic deficit that may precede AD and lay its foundations. We focused on NCX1 and NCX3, since they are crucially involved in pathological settings where metabolic derangements have a prominent role. We have recently characterized the existence of a functional connection between NCX1 activity and metabolic substrates utilization, with particular reference to glutamate, in different in vitro pathological settings, such as cardiac and neuronal ischemia, PD and AD [36,40]. Under our culture conditions SH-SY5Y cells do not express the NCX2 isoform, either when undifferentiated or after differentiation with RA [42]. Therefore, the sequential silencing of the two isoforms allowed us to specifically assess the role of each one in this specific experimental setting. In this context, we found an isoform-specific contribution to the GA-induced cell damage. In particular, the knocking-down of NCX3 was capable of halting the neuronal damage progression, improving cell viability along with a full recovery of ATP levels and of mitochondrial ROS production to control values. In addition, NCX3 silencing completely counteracted the incremental shifts of Aβ and pTau levels provoked by GA. In stark contrast, NCX1 silencing solely prevented the GA-dependent reduction in ATP levels, having neutral effects on the other variables and markers used for cell damage and AD-related injury. We found an overall significant reduction in exchangers’ activity, monitored as Ca^2+^ uptake, following GA treatment. The activity was normalized towards control levels when NCX3 expression was silenced. Conversely, the knocking- down of NCX1 was totally ineffective. It is interesting to note that these effects were not related to any changes in the expression of the exchangers, suggesting that the derangement of Ca^2+^ levels following NCX reverse mode stimulation were mainly due to a modification of the transport activity rather than to a decreased expression of the exchangers, or alternatively, to a redistribution of the surface fraction of the exchangers; both hypotheses deserve further evaluations. A similar result has been observed in RA-differentiated SH-SY5Y cells exposed to hypoxia/reoxygenation (H/R) insult, which alters cell metabolism as well [40]. It is possible to speculate that this overall modification of the exchangers activity may follow a global ionic dysregulation resulting from the drop of ATP intracellular content. As aforementioned, the expression pattern of the exchangers in the SH-SY5Y cell line allowed us to dissect the role of NCX1 and NCX3. The knocking-down of NCX3 implied that NCX1 was the only “available” isoform potentially capable of modulating Ca^2+^ levels in this specific context; conversely, the knocking-down of NCX1 allowed NCX3 to mainly handle Ca^2+^ dynamics. In this view, data obtained by knocking-down NCX3 disclosed a prominent and detrimental role of this isoform, as its elimination positively affected GA-related cell outcomes. It seems more likely to hypothesize a negative role of NCX3 rather than a protective one of NCX1, since the knocking-down of the latter did not produce any changes in GA-related effects; in particular, we did not observe a worsening of the overall cellular response to GA, suggesting a neutral role of NCX1. This observation is in line with the neutral role of NCX1 in the above-mentioned H/R setting [40]. The only cellular response that seemed to rely on both NCX1 and NCX3 was the production of ATP. Consistent with this hypothesis, the use of the pharmacological inhibitor SN6, which is slightly selective for the NCX1 isoform [43], did not produce any effect on GA-related outcomes, except for the increase in ATP levels.

It must be highlighted that the experimental setting used in this work attempts to reproduce a condition that precedes the manifestation of the disease, rather than a situation where cells already show the main AD hallmarks; therefore, our data are not in conflict with the available literature on the role of NCX3 in AD. To date, different studies provide evidence for a protective role of NCX3 in various AD models [33,35,45,46,47]. First, in 1991, Colvin and colleagues observed an increase in NCX activity in surviving neurons of AD patients, suggesting that it may confer protection against AD neurodegenerative processes [45]. Later on, it has been observed that Aβ overproduction may promote the calpain-mediated cleavage of NCX3 in AD brain, supporting the contribution of NCX3 in limiting the intracellular Ca^2+^ overload and preserving cell viability [35]. Further, the reduction of both NCX2 and NCX3 expression seems to be related to cognitive decline in hippocampal CA1 neurons of APP transgenic mice [46]. All the above-mentioned studies focused on investigating the role of NCX3 during a clearly established neuronal damage, while our aim was to gain further insights on the mechanisms underlying the early stage of AD, often characterized by a defective metabolism as a main hallmark. In line with our findings, available data demonstrate that in pathological settings where metabolic dysfunctions prevail, NCX3 may play a central role in promoting neurotoxicity [32,34]. For instance, in cortical neurons subjected to severe transient ischemic and excitotoxic insults, NCX3 knocking-down is shown to ameliorate cell viability. Moreover, a neurotoxic role of NCX3 was observed in BHK cells stably transfected with NCX3 exposed to the fungicide ziram, a pesticide known to increase the risk of developing PD by altering the cellular redox status [48,49] and disrupting mitochondrial activity. In particular, BHK-NCX3 were more vulnerable to ziram-induced intracellular Ca^2+^ overload and mitochondrial dysfunction than BHK wild type cells, suggesting that NCX3 leads to a worsening of cell damage during ziram neurotoxicity [34].

It should be emphasized that the data related to NCX activity are widely debated and strongly linked to the specific context the exchangers are working in. The proposed model is based on the use of SH-SY5Y, which is a human neuroblastoma cell line; here, we observed a global decline of the NCXs’ activity. However, in a previous study, we observed that in primary rat cortical neurons, GA induced an increase in the overall NCX activity [36]. The apparent discrepancy can be explained considering two main issues: (1) the different nature of the experimental models (human vs. rat); (2) the expression of NCX2 isoform in rat cortical neurons. Further studies are needed to better clarify the actual contribution of each single NCX isoform in this setting, which is characterized by several complexities. Despite the different impact of GA on the exchangers’ activity, it seems quite obvious that such a metabolic alteration can perturb intracellular Ca^2+^ dynamics, with deleterious consequences on neuronal activities, ranging from mitochondrial dysfunction, oxidative stress, deposition of Aβ, and hyperphosphorylation of Tau protein. While RA-differentiated SH-SY5Y cells are a simple and easy to handle in vitro model, we have to consider that it does not involve glial cells, which are well known to sustain neuronal functions, with main regard to the metabolism and substrates utilization [50]. A denser accumulation of astrocytes colocalized with AGEs has been observed in patients with AD [51,52,53], suggesting a role of surrounding astrocytes in triggering a cascade of events that may affect the neighboring neurons [54], preparing for a shift in perspective of the disease pathogenesis that deserve further investigations.

In conclusion, our work demonstrated that: (1) NCX1 and NCX3 can operate in different ways strictly depending on the specific environmental settings, and that (2) NCX3 has a strong impact on GA-induced cytotoxicity, since NCX3 knocking-down appears to increase ATP production, rescue the intracellular Ca^2+^ perturbation, reduce the accumulation of both Aβ and pTau, and mitigate oxidative damage thus promoting cell survival. Overall, these findings highlight the importance of NCX3 in setting the conditions to trigger neurodegeneration, providing a potential target for slowing down or preventing AD.

## Data Availability

The data presented in this study are available on request from the corresponding author.

## Supplementary Materials

The following supporting information can be downloaded at: https://www.mdpi.com/article/10.3390/biology12071005/s1, Figure S1: Expression of NCX1 and NCX3 in RA-differentiated SH-SY5Y cells after NCX3 and NCX1 silencing and GA-challenge.

## References

[1] GBD 2019 Dementia Forecasting Collaborators (2022). Estimation of the global prevalence of dementia in 2019 and forecasted prevalence in 2050: An analysis for the Global Burden of Disease Study 2019. Lancet Public Health.

[2] van der Flier W.M., de Vugt M.E., Smets E.M.A., Blom M., Teunissen C.E. (2023). Towards a future where Alzheimer’s disease pathology is stopped before the onset of dementia. Nat. Aging.

[3] Pimplikar S.W. (2014). Neuroinflammation in Alzheimer’s disease: From pathogenesis to a therapeutic target. J. Clin. Immunol..

[4] Guo T., Zhang D., Zeng Y., Huang T.Y., Xu H., Zhao Y. (2020). Molecular and cellular mechanisms underlying the pathogenesis of Alzheimer’s disease. Mol. Neurodegener..

[5] Yan X., Hu Y., Wang B., Wang S., Zhang X. (2020). Metabolic Dysregulation Contributes to the Progression of Alzheimer’s Disease. Front. Neurosci..

[6] Hammond T.C., Lin A.L. (2022). Glucose Metabolism is a Better Marker for Predicting Clinical Alzheimer’s Disease than Amyloid or Tau. J. Cell Immunol..

[7] Hoyer S. (1985). The effect of age on glucose and energy metabolism in brain cortex of rats. Arch. Gerontol. Geriatr..

[8] Butterfield D.A., Halliwell B. (2019). Oxidative stress, dysfunctional glucose metabolism and Alzheimer disease. Nat. Rev. Neurosci..

[9] Strom A., Iaccarino L., Edwards L., Lesman-Segev O.H., Soleimani-Meigooni D.N., Pham J., Baker S.L., Landau S.M., Jagust W.J., Miller B.L. (2022). Cortical hypometabolism reflects local atrophy and tau pathology in symptomatic Alzheimer’s disease. Brain.

[10] Calsolaro V., Edison P. (2016). Alterations in Glucose Metabolism in Alzheimer’s Disease. Recent Pat. Endocr. Metab. Immune Drug Discov..

[11] Dewanjee S., Chakraborty P., Bhattacharya H., Chacko L., Singh B., Chaudhary A., Javvaji K., Pradhan S.R., Vallamkondu J., Dey A. (2022). Altered glucose metabolism in Alzheimer’s disease: Role of mitochondrial dysfunction and oxidative stress. Free Radic. Biol. Med..

[12] Cisternas P., Zolezzi J.M., Martinez M., Torres V.I., Wong G.W., Inestrosa N.C. (2019). Wnt-induced activation of glucose metabolism mediates the in vivo neuroprotective roles of Wnt signaling in Alzheimer disease. J. Neurochem..

[13] An Y., Varma V.R., Varma S., Casanova R., Dammer E., Pletnikova O., Chia C.W., Egan J.M., Ferrucci L., Troncoso J. (2018). Evidence for brain glucose dysregulation in Alzheimer’s disease. Alzheimers Dement..

[14] Piccirillo S., Preziuso A., Amoroso S., Serfilippi T., Miceli F., Magi S., Lariccia V. (2022). A new K^+^ channel-independent mechanism is involved in the antioxidant effect of XE-991 in an in vitro model of glucose metabolism impairment: Implications for Alzheimer’s disease. Cell Death Discov..

[15] Mattson M.P., Barger S.W., Cheng B., Lieberburg I., Smith-Swintosky V.L., Rydel R.E. (1993). β-Amyloid precursor protein metabolites and loss of neuronal Ca^2+^ homeostasis in Alzheimer’s disease. Trends Neurosci..

[16] Nikoletopoulou V., Tavernarakis N. (2012). Calcium homeostasis in aging neurons. Front. Genet..

[17] Marambaud P., Dreses-Werringloer U., Vingtdeux V. (2009). Calcium signaling in neurodegeneration. Mol. Neurodegener..

[18] Guan P.P., Cao L.L., Wang P. (2021). Elevating the Levels of Calcium Ions Exacerbate Alzheimer’s Disease via Inducing the Production and Aggregation of β-Amyloid Protein and Phosphorylated Tau. Int. J. Mol. Sci..

[19] Webber E.K., Fivaz M., Stutzmann G.E., Griffioen G. (2023). Cytosolic calcium: Judge, jury and executioner of neurodegeneration in Alzheimer’s disease and beyond. Alzheimers Dement..

[20] Demuro A., Parker I., Stutzmann G.E. (2010). Calcium signaling and amyloid toxicity in Alzheimer disease. J. Biol. Chem..

[21] Itkin A., Dupres V., Dufrene Y.F., Bechinger B., Ruysschaert J.M., Raussens V. (2011). Calcium ions promote formation of amyloid β-peptide (1–40) oligomers causally implicated in neuronal toxicity of Alzheimer’s disease. PLoS ONE.

[22] Green K.N., LaFerla F.M. (2008). Linking calcium to Aβ and Alzheimer’s disease. Neuron.

[23] Ahmad A., Muzaffar M., Ingram V.M. (2009). Ca^2+^, within the physiological concentrations, selectively accelerates Aβ_42_ fibril formation and not Aβ_40_ in vitro. Biochim. Biophys. Acta.

[24] Ge M., Zhang J., Chen S., Huang Y., Chen W., He L., Zhang Y. (2022). Role of Calcium Homeostasis in Alzheimer’s Disease. Neuropsychiatr. Dis. Treat..

[25] Rodrigues T., Piccirillo S., Magi S., Preziuso A., Dos Santos Ramos V., Serfilippi T., Orciani M., Maciel Palacio Alvarez M., Luis Dos Santos Tersariol I., Amoroso S. (2022). Control of Ca^2+^ and metabolic homeostasis by the Na^+^/Ca^2+^ exchangers (NCXs) in health and disease. Biochem. Pharmacol..

[26] Blaustein M.P., Lederer W.J. (1999). Sodium/calcium exchange: Its physiological implications. Physiol. Rev..

[27] Lariccia V., Piccirillo S., Preziuso A., Amoroso S., Magi S. (2020). Cracking the code of sodium/calcium exchanger (NCX) gating: Old and new complexities surfacing from the deep web of secondary regulations. Cell Calcium.

[28] Quednau B.D., Nicoll D.A., Philipson K.D. (1997). Tissue specificity and alternative splicing of the Na^+^/Ca^2+^ exchanger isoforms NCX1, NCX2, and NCX3 in rat. Am. J. Physiol..

[29] Khananshvili D. (2013). The SLC8 gene family of sodium-calcium exchangers (NCX)—Structure, function, and regulation in health and disease. Mol. Asp. Med..

[30] Pannaccione A., Piccialli I., Secondo A., Ciccone R., Molinaro P., Boscia F., Annunziato L. (2020). The Na^+^/Ca^2+^ exchanger in Alzheimer’s disease. Cell Calcium.

[31] Bano D., Young K.W., Guerin C.J., Lefeuvre R., Rothwell N.J., Naldini L., Rizzuto R., Carafoli E., Nicotera P. (2005). Cleavage of the plasma membrane Na^+^/Ca^2+^ exchanger in excitotoxicity. Cell.

[32] Cross J.L., Meloni B.P., Bakker A.J., Sokolow S., Herchuelz A., Schurmans S., Knuckey N.W. (2009). Neuronal injury in NCX3 knockout mice following permanent focal cerebral ischemia and in NCX3 knockout cortical neuronal cultures following oxygen-glucose deprivation and glutamate exposure. J. Exp. Stroke Transl. Med..

[33] Sokolow S., Luu S.H., Headley A.J., Hanson A.Y., Kim T., Miller C.A., Vinters H.V., Gylys K.H. (2011). High levels of synaptosomal Na^+^-Ca^2+^ exchangers (NCX1, NCX2, NCX3) co-localized with amyloid-β in human cerebral cortex affected by Alzheimer’s disease. Cell Calcium.

[34] Jin J., Lao A.J., Katsura M., Caputo A., Schweizer F.E., Sokolow S. (2014). Involvement of the sodium-calcium exchanger 3 (NCX3) in ziram-induced calcium dysregulation and toxicity. Neurotoxicology.

[35] Atherton J., Kurbatskaya K., Bondulich M., Croft C.L., Garwood C.J., Chhabra R., Wray S., Jeromin A., Hanger D.P., Noble W. (2014). Calpain cleavage and inactivation of the sodium calcium exchanger-3 occur downstream of Aβ in Alzheimer’s disease. Aging Cell.

[36] Magi S., Piccirillo S., Maiolino M., Lariccia V., Amoroso S. (2020). NCX1 and EAAC1 transporters are involved in the protective action of glutamate in an in vitro Alzheimer’s disease-like model. Cell Calcium.

[37] Piccirillo S., Magi S., Preziuso A., Castaldo P., Amoroso S., Lariccia V. (2020). Gateways for Glutamate Neuroprotection in Parkinson’s Disease (PD): Essential Role of EAAT3 and NCX1 Revealed in an In Vitro Model of PD. Cells.

[38] Maiolino M., Castaldo P., Lariccia V., Piccirillo S., Amoroso S., Magi S. (2017). Essential role of the Na^+^-Ca^2+^ exchanger (NCX) in glutamate-enhanced cell survival in cardiac cells exposed to hypoxia/reoxygenation. Sci. Rep..

[39] Castaldo P., Macri M.L., Lariccia V., Matteucci A., Maiolino M., Gratteri S., Amoroso S., Magi S. (2017). Na^+^/Ca^2+^ exchanger 1 inhibition abolishes ischemic tolerance induced by ischemic preconditioning in different cardiac models. Eur. J. Pharmacol..

[40] Piccirillo S., Castaldo P., Macri M.L., Amoroso S., Magi S. (2018). Glutamate as a potential “survival factor” in an in vitro model of neuronal hypoxia/reoxygenation injury: Leading role of the Na^+^/Ca^2+^ exchanger. Cell Death Dis..

[41] Shenoda B. (2015). The role of Na^+^/Ca^2+^ exchanger subtypes in neuronal ischemic injury. Transl. Stroke Res..

[42] Iwamoto T., Kita S. (2006). YM-244769, a novel Na^+^/Ca^2+^ exchange inhibitor that preferentially inhibits NCX3, efficiently protects against hypoxia/reoxygenation-induced SH-SY5Y neuronal cell damage. Mol. Pharmacol..

[43] Iwamoto T., Inoue Y., Ito K., Sakaue T., Kita S., Katsuragi T. (2004). The exchanger inhibitory peptide region-dependent inhibition of Na^+^/Ca^2+^ exchange by SN-6 [2-[4-(4-nitrobenzyloxy)benzyl]thiazolidine-4-carboxylic acid ethyl ester], a novel benzyloxyphenyl derivative. Mol. Pharmacol..

[44] Koriyama Y., Furukawa A., Muramatsu M., Takino J., Takeuchi M. (2015). Glyceraldehyde caused Alzheimer’s disease-like alterations in diagnostic marker levels in SH-SY5Y human neuroblastoma cells. Sci. Rep..

[45] Colvin R.A., Bennett J.W., Colvin S.L., Allen R.A., Martinez J., Miner G.D. (1991). Na^+^/Ca^2+^ exchange activity is increased in Alzheimer’s disease brain tissues. Brain Res..

[46] Moriguchi S., Kita S., Fukaya M., Osanai M., Inagaki R., Sasaki Y., Izumi H., Horie K., Takeda J., Saito T. (2018). Reduced expression of Na^+^/Ca^2+^ exchangers is associated with cognitive deficits seen in Alzheimer’s disease model mice. Neuropharmacology.

[47] Pannaccione A., Secondo A., Molinaro P., D’Avanzo C., Cantile M., Esposito A., Boscia F., Scorziello A., Sirabella R., Sokolow S. (2012). A new concept: Aβ_1-42_ generates a hyperfunctional proteolytic NCX3 fragment that delays caspase-12 activation and neuronal death. J. Neurosci..

[48] Kanemoto-Kataoka Y., Oyama K., Oyama T.M., Ishibashi H., Oyama Y. (2018). Ziram, a dithiocarbamate fungicide, exhibits pseudo-cytoprotective actions against oxidative stress in rat thymocytes: Possible environmental risks. Environ. Res..

[49] Martin C.A., Myers K.M., Chen A., Martin N.T., Barajas A., Schweizer F.E., Krantz D.E. (2016). Ziram, a pesticide associated with increased risk for Parkinson’s disease, differentially affects the presynaptic function of aminergic and glutamatergic nerve terminals at the Drosophila neuromuscular junction. Exp. Neurol..

[50] Jha M.K., Morrison B.M. (2018). Glia-neuron energy metabolism in health and diseases: New insights into the role of nervous system metabolic transporters. Exp. Neurol..

[51] Wong A., Luth H.J., Deuther-Conrad W., Dukic-Stefanovic S., Gasic-Milenkovic J., Arendt T., Munch G. (2001). Advanced glycation endproducts co-localize with inducible nitric oxide synthase in Alzheimer’s disease. Brain Res..

[52] Luth H.J., Ogunlade V., Kuhla B., Kientsch-Engel R., Stahl P., Webster J., Arendt T., Munch G. (2005). Age- and stage-dependent accumulation of advanced glycation end products in intracellular deposits in normal and Alzheimer’s disease brains. Cereb. Cortex.

[53] Prasad K. (2019). AGE-RAGE stress: A changing landscape in pathology and treatment of Alzheimer’s disease. Mol. Cell Biochem..

[54] Sollvander S., Nikitidou E., Brolin R., Soderberg L., Sehlin D., Lannfelt L., Erlandsson A. (2016). Accumulation of amyloid-β by astrocytes result in enlarged endosomes and microvesicle-induced apoptosis of neurons. Mol. Neurodegener..

