# Supplementary material for: Exploring the Role of NCX1 and NCX3 in an In Vitro Model of Metabolism Impairment: Potential Neuroprotective Targets for Alzheimer’s Disease
Source: Biology (Basel). 2023 Jul 14;12(7):1005. doi: 10.3390/biology12071005 (PMC10376230; doi:10.3390/biology12071005)
Supplement: Supplementary file 1 [file biology-12-01005-s001.zip › biology-2465038-supplementary.pdf]

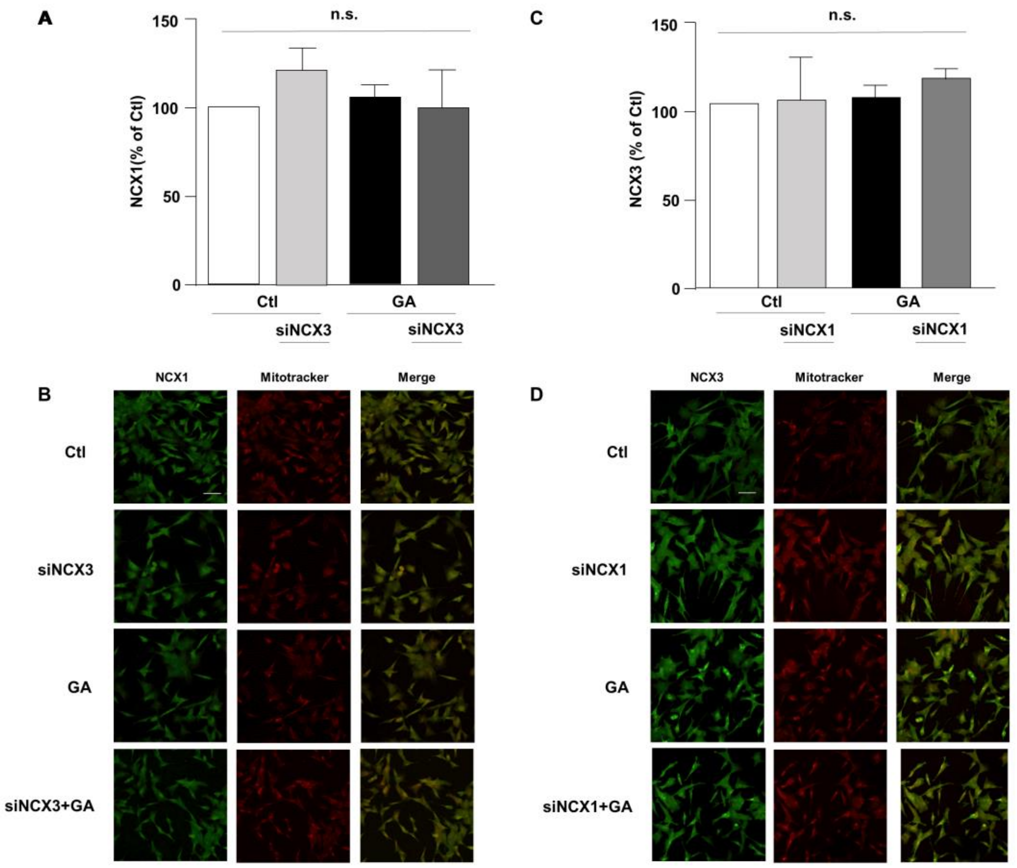

Figure S1. Expression of NCX1 and NCX3 in RA-differentiated SH-SY5Y cells after NCX3 and NCX1 silencing and GA-challenge.
